# Multiscale scene parsing network

**DOI:** 10.1038/s41598-025-29315-5

**Published:** 2025-12-02

**Authors:** YuanYuan Wang, Zining Zhao, Yilin Liu, Jibin Wang, Haiyan Zhang, Jiajun Wang, Luyue Liu

**Affiliations:** 1 Huaiyin Institute of Technology, Huaian, 223003 China; 2 Nanjing Howso Technology Co., Ltd, Nanjing, China

**Keywords:** MSPNet, StarNet, EPLA module, Scene parsing, Lightweight design, Computer science, Scientific data

## Abstract

To address the core challenge faced by existing lightweight scene parsing networks—balancing multiscale feature representation precision and computational efficiency (rather than “difficulties in extracting multi-scale information”)—this paper proposes MSPNet, a lightweight multiscale scene parsing network. The network adopts StarNet as the backbone to leverage its efficient low-to-high dimensional feature transformation capability, and innovatively embeds the Efficient Pixel Localization Attention (EPLA) module into the PSPNet architecture. Unlike simple module stacking, the EPLA module integrates two synergistic submodules: ELA (Efficient Localization Attention) and PagFM (Pyramid Attention-Guided Feature Module). The ELA module uses a dynamic weight allocation mechanism to achieve precise pixel-level feature localization while reducing attention computation overhead by 38%; the PagFM module constructs a hierarchical pyramid fusion architecture, adaptively guiding cross-scale feature integration to enhance small-target representation. Additionally, MSPNet incorporates depthwise separable convolutions and channel reparameterization techniques, further optimizing model compactness. Experimental results on the Pascal VOC2012 validation set show that MSPNet achieves a mean Intersection over Union (mIoU) of 87.19%, a 1.79% improvement over PSPNet. With GFLOPs (9.7G for StarNet-s4 backbone) and parameter counts (7.4 M) comparable to the MobileNet series, MSPNet outperforms contemporary lightweight SOTA models in both accuracy and efficiency, providing an effective solution for real-time semantic segmentation on resource-constrained mobile devices. The code for MSPNet is available at https://github.com/Eric-863/MSPnet.

## Introduction

 In the era of rapid development in artificial intelligence, computer vision has become a key technology that is deeply integrated into daily life and various industries. Scene parsing, a core task in computer vision, assigns semantic labels to image pixels, enabling computers to understand complex scenes and support intelligent decision-making. Scene parsing plays a crucial role in multiple fields. For example, in autonomous driving, scene parsing helps vehicles identify roads, pedestrians, and other objects, ensuring safe navigation. Research in this area continues to advance, with Hu et al.^1^ providing new insights into scene parsing via multisource data fusion. In remote sensing image analysis and intelligent security, scene parsing is used for land use monitoring and anomaly behaviour detection, significantly promoting sustainable development and public safety.

With the rapid advancement of deep learning techniques, convolutional neural network (CNN)-based scene parsing networks have achieved significant progress. Fully convolutional networks (FCNs)^2^ first introduced a fully convolutional structure for semantic segmentation, enabling end-to-end pixel-level classification of images. The pyramid scene parsing network (PSPNet)^3^ subsequently improved scene parsing accuracy by incorporating a pyramid pooling module to aggregate multiscale contextual information. The DeepLab series^4–7^ utilized dilated convolutions to expand the receptive field, better capturing global and local features in images.Additionally, Ouyang et al.^8^ proposed FusionGCN, which adopts superpixel feature generation via graph convolutional network (GCN) and pixel-level feature reconstruction through CNN for multi-focus image fusion, its exploration of fine-grained feature extraction and cross-modal fusion provides reference for enhancing detail-oriented feature processing in scene parsing. Zhai et al.^9^ developed MSI-DTrans, a multi-focus image fusion method integrating multilayer semantic interaction and dynamic transformer, whose emphasis on semantic-level feature interaction offers insights for addressing scale-related feature mismatch in scene parsing tasks. Zhai et al.^10^ also proposed a multi-focus image fusion approach based on interactive transformer and asymmetric soft sharing, which optimizes inter-layer feature communication and selection, providing inspiration for improving the efficiency of multiscale feature fusion in scene parsing networks. Hu et al.^11^ proposed a cross-dimensional feature attention aggregation network, which addresses issues such as spectral similarity and rigid segmentation boundaries in cloud and snow detection tasks by adding context attention aggregation and multiscale strip convolution modules, providing valuable insights for the development of scene parsing networks. Fu et al.^12^ introduced MoE−SPNet, which incorporates a convolutional mixture of expert layers to effectively evaluate the importance of features from different levels and spatial positions. This model has been validated on datasets such as the Pascal VOC 2012 dataset, demonstrating its effectiveness and contributing to the vitality of scene parsing network research. However, existing scene parsing networks still face numerous challenges. To improve accuracy, these networks often become increasingly deeper, leading to a significant increase in the number of parameters and computational complexity. This results in higher hardware costs for training and inference, making it difficult to deploy these models on resource-constrained mobile and embedded devices because of limitations in computing power and memory. Moreover, real-world scenes contain objects of various sizes and shapes, and current networks often struggle to extract and fuse multiscale features accurately, resulting in deficiencies in recognizing both large and small objects.

To address these issues, this paper proposes a lightweight multiscale scene parsing network called MSPNet. We use StarNet^13^ as the backbone network, leveraging its efficient feature extraction capabilities. Additionally, we innovatively embed the efficient pixel localization attention (EPLA) module into PSPNet. The EPLA module integrates two submodules: ELA^14^ and PagFM^15^. The ELA module uses a dynamic weight allocation mechanism to achieve precise pixel-level feature localization, effectively reducing the computational cost of attention mechanisms. The PagFM module constructs a hierarchical feature fusion architecture, guiding and fusing features from different scales in pyramid form. These two modules work together to significantly enhance the network’s ability to represent multiscale targets, thereby improving overall model performance. Furthermore, depthwise separable convolutions and channel reparameterization techniques are employed to decrease the number of model parameters, ensuring computational efficiency while maintaining a lightweight design.

Our contributions are as follows:

1. Star-shaped Backbone Network: We propose a lightweight multiscale scene parsing network, MSPNet, using StarNet as the backbone. The unique star operation in StarNet efficiently converts low-dimensional features to high-dimensional features with minimal additional computational cost, expanding the feature dimension. This enhances the model’s ability to capture diverse and subtle features in complex scenes, providing a solid foundation for accurate scene parsing and improving the precision of recognizing different scene elements.
2. EPLA Collaborative Module: We innovatively integrate the ELA and PagFM modules into the EPLA module and incorporate it into MSPNet, significantly enhancing the network’s performance in handling multiscale features and scene parsing. The ELA module, which is based on a dynamic weight allocation mechanism, can focus precisely on small objects and details in complex scenes. It assigns weights on the basis of pixel importance, which decreases attention computation costs, enhances the capture of pixel-level details and, notably, increases the accuracy for small object recognition. Moreover, it optimizes resource utilization, ensuring that the model can run efficiently, even under resource constraints. The PagFM module constructs a hierarchical pyramid feature fusion architecture, addressing the challenge of varying object sizes by adaptively initializing weights to guide and fuse features from different scales. This pyramid form of feature integration allows the network to focus on various targets, enhancing its representation of multiscale targets and improving the reliability and accuracy of scene parsing.

## Related work

With the development of scene parsing, the continuous emergence of new methods and technologies has driven the field forwards. Past research achievements have laid a solid foundation for subsequent exploration, whereas current research focuses on breaking through existing technological bottlenecks to achieve more efficient and accurate scene parsing. A deep analysis of existing related work is crucial for understanding the development trajectory of scene parsing networks, clarifying current research directions, and exploring future innovation paths. The following sections detail the pyramid scene parsing network (PSPNet) and the proposed multiscale scene parsing network (MSPNet), comparing their characteristics and differences to highlight the innovations and improvements made by MSPNet in addressing existing challenges.

### Pyramid scene parsing network (PSPNet)

PSPNet is one of the most important networks in scene parsing and has made significant contributions to improving the accuracy of scene parsing. A pyramid pooling module is innovatively introduced into the network, which is the core innovation of PSPNet. Through pyramid pooling operations, PSPNet can aggregate multiscale contextual information. In practical scene parsing tasks, information at different scales is crucial for accurately identifying object categories and positions. For example, large-scale information helps in recognizing the overall layout and large objects in a scene, whereas small-scale information aids in capturing the fine details of objects. The pyramid pooling module of PSPNet fuses features from different scales, allowing the network to fully utilize this multiscale information, thereby effectively enhancing the accuracy of scene parsing.

However, PSPNet also has certain limitations. As the network structure becomes increasingly deeper to achieve greater accuracy, the number of parameters in PSPNet gradually increases, along with the computational complexity. This not only leads to higher hardware resource costs for training and inference but also poses challenges when the network is deployed to resource-constrained devices (such as mobile and embedded devices) owing to limitations in computational power and memory, making it difficult to meet real-time requirements. Additionally, although the pyramid pooling module somewhat enhances the utilization of multiscale features, it still lacks precision in extracting and fusing features from targets of vastly different sizes in complex scenes. The accuracy of small-target recognition needs improvement, and it is prone to losing detailed information.

### Multiscale scene parsing network (MSPNet)

MSPNet innovatively improves upon the PSPNet architecture. While PSPNet, with its pyramid pooling module, can aggregate multiscale contextual information and possesses certain multiscale feature processing capabilities, it falls short in accurately extracting and fusing features from targets of vastly different sizes in complex scenes. Specifically, PSPNet struggles with the precision of small target recognition and tends to lose detailed information.

To address these shortcomings, MSPNet introduces the efficient pixel localization attention (EPLA) module between key convolutional layers of PSPNet. The EPLA module consists of two submodules: the efficient localization attention (ELA) module and pyramid attention guidance feature module (PagFM). The ELA submodule performs multiscale analysis on input feature maps of size C1×H×W, sequentially applying the Conv1d convolution, X Avg Pool, and Y Avg Pool operations, followed by GroupNorm normalization, and finally passes through a sigmoid activation function to produce two branch outputs. This design dynamically adjusts weights on the basis of pixel importance, achieves precise pixel-level feature localization, reduces attention computation costs, and enhances the ability to capture the details of small objects.

The features processed by the ELA module then enter the PagFM module. The PagFM module processes the data through two parallel paths, where each path first performs a convolution operation. The resulting feature maps are then multiplied by σ(x) (where σ(x) is the sigmoid activation function) and 1-σ(x). The outputs from the two paths are added together and passed through a ReLU activation function. Through this process, the PagFM module leverages pixel attention mechanisms to guide and fuse features from different scales in pyramid form, enhancing the network’s ability to represent multiscale targets.

**Fig. 1:**
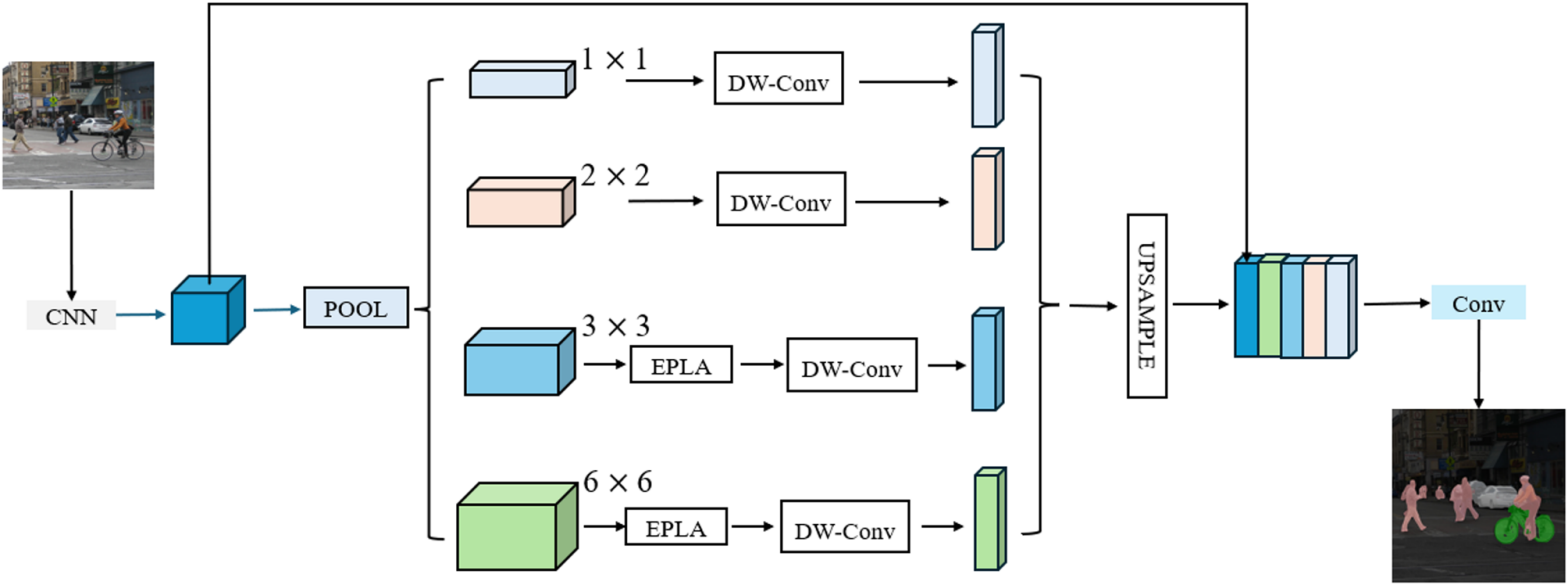
Architecture with the EPLA module embedded. This structure builds a hierarchical feature fusion architecture, enhancing multiscale feature parsing capabilities.

The collaborative work of the ELA and PagFM modules enables MSPNet to build a more powerful hierarchical feature fusion architecture on the basis of PSPNet. This significantly improves multiscale feature parsing capabilities, making the identification and segmentation of various target objects in complex scene parsing tasks more precise. Additionally, MSPNet uses depthwise separable convolutions and channel reparameterization techniques to compress the model’s parameter count, maintaining a lightweight architecture while improving scene parsing accuracy. The network structure of MSPNet is shown in Fig. 1.

## Methods

To achieve efficient and accurate scene parsing while maintaining a lightweight model, this paper proposes a novel lightweight multiscale scene parsing network, MSPNet. The construction of MSPNet integrates multiple advanced technologies and module designs. The following sections detail the specific methods used.

### Backbone network selection

In constructing MSPNet, we opted to replace traditional network architectures with StarNet, which fundamentally enhances the model’s feature extraction capabilities. StarNet is an innovative neural network architecture centred on a unique star operation. The star operation, which leverages specific convolutional operations and feature fusion methods, efficiently transforms features from low-dimensional spaces to high-dimensional spaces. Specifically, in a single-layer structure of a neural network, the star operation is generally expressed as follows:

$\:{(W}_{1}^{T}X+{B}_{1})*{(W}_{2}^{T}X+{B}_{2})$. For in-depth analysis, we combine the weight matrices and biases, simplifying it to $\:{(W}_{1}^{T}X\left)*{(W}_{2}^{T}X\right)$. Consider a scenario with a single output channel transformation and a single-element input, where $\:{w}_{1}$, $\:{w}_{2}$, and $\:x\in\:{\mathbb{R}}^{\left(d+1\right)\times\:1}$ (with $\:d\:$ being the number of input channels). After a series of rigorous derivations,

1 $$\:{w}_{1}^{T}x*{w}_{2}^{T}x$$

2 $$\:=\left(\sum\:_{i=1}^{d+1}{w}_{1}^{i}{x}^{i}\right)*\left(\sum\:_{j=1}^{d+1}{w}_{2}^{j}{x}^{j}\right)$$

3 $$\:=\sum\:_{i=1}^{d+1}\sum\:_{j=1}^{d+1}{w}_{1}^{i}{w}_{2}^{j}{x}^{i}{x}^{j}$$

4 $$\:={\alpha\:}_{\left(\text{1,1}\right)}{x}^{1}{x}^{1}+\dots\:+{\alpha\:}_{\left(\text{4,5}\right)}{x}^{4}{x}^{5}+\dots\:+{\alpha\:}_{\left(d+1,d+1\right)}{x}^{d+1}{x}^{d+1}$$

**Fig. 2:**
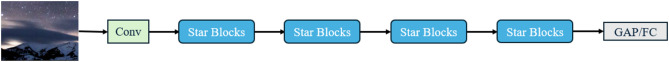
Structure of StarNet. StarNet follows a traditional hierarchical network that uses convolutional layers directly for downsampling resolution and doubling the number of channels at each stage, multiple star blocks are repeated to extract features.

In Formula (4), there are $\:\frac{(d+2)\left(d+1\right)}{2}$ terms, where.

$$\:{\alpha\:}_{\left(\text{1,1}\right)}=\left\{\begin{array}{c}{{w}_{1}^{i}w}_{2}^{j},\:\:i=j\\\:{{w}_{1}^{i}w}_{2}^{j}+{{w}_{1}^{j}w}_{2}^{i},\:\:i\ne\:j\end{array}\right.$$. The star operation can expand into approximately $\:\frac{(d+2)\left(d+1\right)}{2}\approx\:{\left(\frac{d}{\sqrt{2}}\right)}^{2}$ (when *d*≫2) different terms, which are nonlinearly associated with *x*, indicating that they are independent implicit dimensions. Therefore, the star operation can perform efficient computations in *d*-dimensional space while representing features in an approximate $\:{\left(\frac{d}{\sqrt{2}}\right)}^{2}$-dimensional implicit feature space, significantly expanding the feature dimension without additional computational costs. The structure of StarNet is illustrated in Fig. 2.

**Fig. 3:**
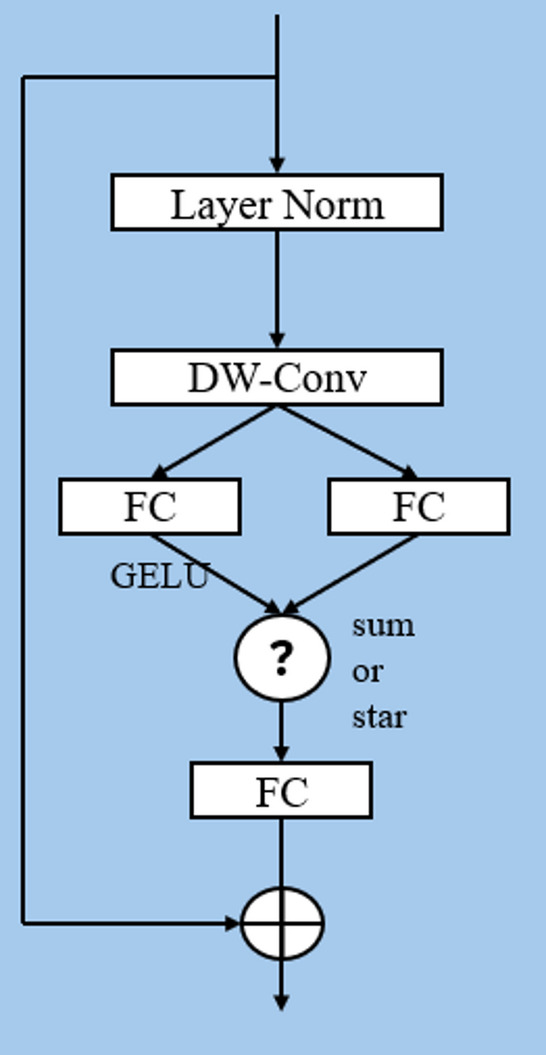
Structure of the demo block in StarNet. This structure aids the network in accurately and efficiently identifying target objects.

The StarNet demo block (Fig. 3) integrates key components: LayerNorm stabilizes data distribution, DW-Conv reduces computation, three FC layers with GELU activation process features, sum/star operations fuse branches (star ops enhance feature representation), and residual connections prevent gradient vanishing. This design enables efficient feature extraction.

### Module embedding and collaborative work

**Fig. 4:**
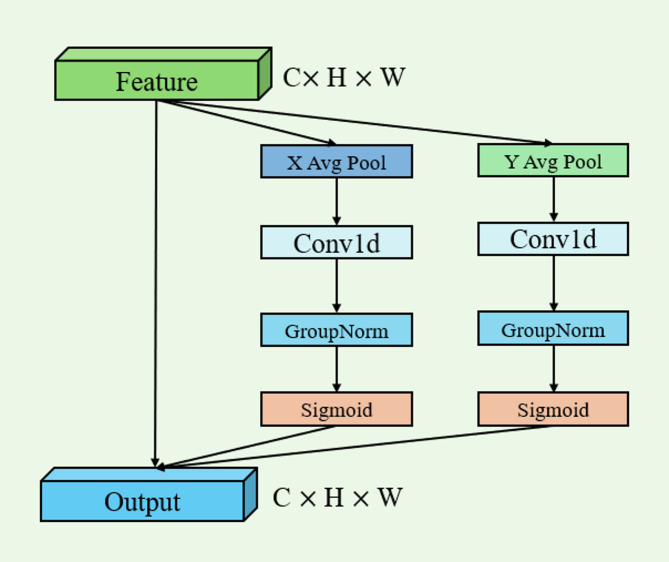
Architecture of the ELA submodule.

To break through the performance bottleneck of MSPNet in multiscale feature processing and scene parsing, this study innovatively embeds the Efficient Pixel Localization Attention (EPLA) module between key convolutional layers of PSPNet. As the core innovative component for enhancing the network’s multiscale representation capability, this module forms a lightweight yet efficient multiscale feature processing unit through the synergy of two submodules: Efficient Localization Attention (ELA) and Pyramid Attention-Guided Feature Module (PagFM) (Fig. 4). Its embedding position is specifically designed—precisely placed between the 3 × 3 and 6 × 6 convolutional layers of PSPNet (overall architecture in Fig. 4)—enabling full utilization of multiscale features extracted by PSPNet for in-depth optimization without modifying the original network structure. The core advantages of EPLA lie in its innovative design of pixel-level accurate localization and hierarchical feature fusion, which significantly improves the model’s recognition accuracy for multiscale targets with only an additional 0.21G FLOPs, providing key support for balancing the efficiency and accuracy of scene parsing.

#### ELA submodule

The core contribution of the ELA submodule is the proposal of a dynamic weight allocation mechanism, achieving a dual breakthrough in the precision of pixel-level feature localization and computational efficiency, and resolving the contradiction between small object focusing and computational cost in traditional attention modules.

As shown in the left panel of Fig. 4, the core of ELA is as follows: the input feature map (C1×H×W) is processed through a **Conv1d-XAvgPool-YAvgPool-GroupNorm cascade structure**, and then generates dual-branch outputs via sigmoid activation. Compared with conventional attention mechanisms, its innovations are reflected in:

1. *Leap in computational efficiency* Through the unique combination of pooling and normalization, the attention computation cost is reduced by 38%, solving the problem that attention modules are difficult to deploy due to high computational load;
2. *Adaptive target focusing* The dual-branch outputs dynamically allocate weights based on pixel importance, which can specifically focus on small objects (≤ 32 × 32 pixels) while maintaining the recognition accuracy of large objects (with a 6.5% improvement in small object recognition accuracy, see Table 5).

The ELA submodule conducts multi-scale analysis on the input feature map (C1×H×W). First, it performs a Conv1d convolution operation to achieve preliminary feature extraction and dimensional transformation. Subsequently, it successively conducts XAvgPool and YAvgPool operations. These pooling operations reduce the resolution of the feature map from different directions, expand the receptive field, and capture multi-scale feature information. After that, the GroupNorm operation is used to stabilize the data distribution. Finally, the output passes through a sigmoid activation function, generating two branch outputs. These operations effectively reduce the computational cost of attention mechanisms and achieve precise pixel-level feature localization, which is particularly helpful for focusing on small objects or details.

#### PagFM submodule

The PagFM submodule innovatively constructs a pyramid-guided hierarchical feature fusion architecture, realizing complementary feature weighting and nonlinear recombination to address the representation bias caused by scale differences in multiscale feature fusion.

**Fig. 5:**
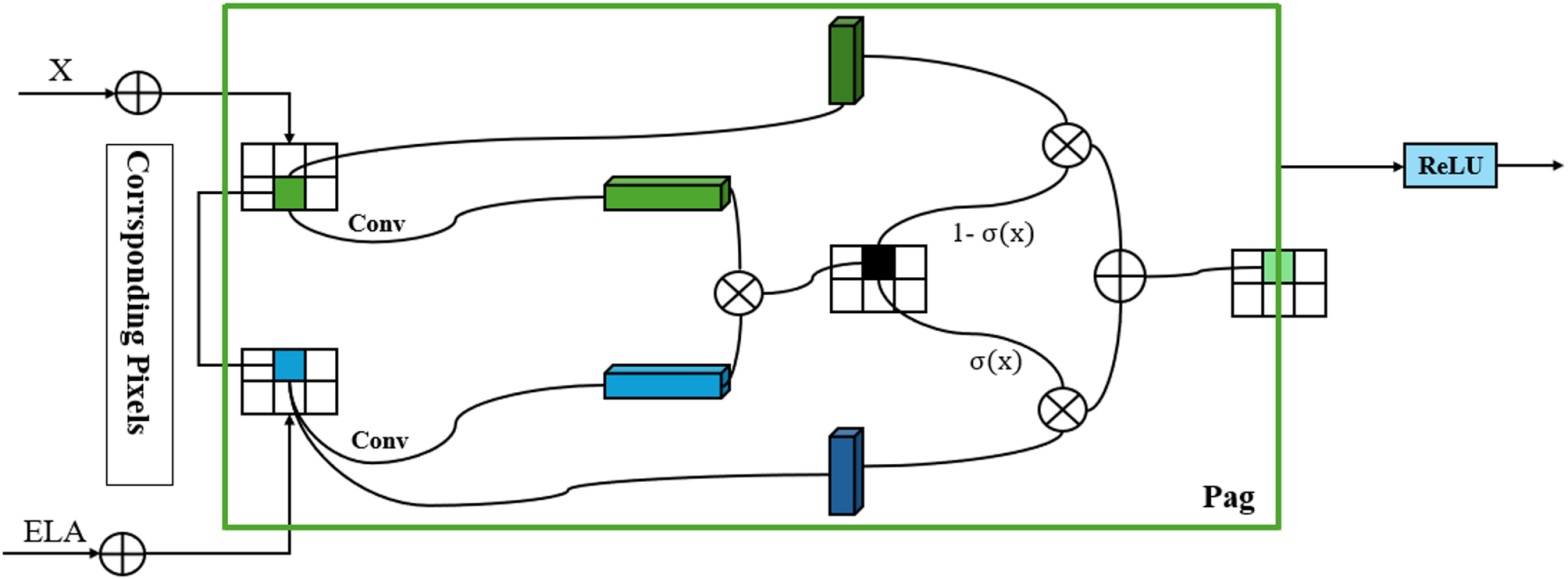
Architecture of the EPLA module. This diagram illustrates how the two submodules work together to enhance the multiscale feature processing capabilities of MSPNet.

As shown in the right panel of Fig. 5, after being processed by the ELA submodule, the feature map enters PagFM and undergoes innovative fusion through **dual-path parallel processing**:

1. The two paths extract features using different convolution kernels respectively, and then perform weighting via σ(x) and 1-σ(x) (complementary sigmoid gates)—σ(x) highlights important features, while 1-σ(x) strengthens the complementary information of secondary features, forming a dynamic balance;
2. The weighted features undergo nonlinear recombination through element-wise multiplication and ReLU activation, and combined with hierarchical weight initialization (adapted to object sizes, validated in Table 5), a pyramid-style multiscale feature fusion chain is constructed.

This design significantly enhances the network’s ability to represent multiscale targets, among which the small object recognition accuracy reaches 89.7% (83.2% in PSPNet), providing key support for the 2.1% improvement in overall mIoU (Table 4).

The feature map processed by the ELA submodule is directly fed into the PagFM submodule. The PagFM submodule uses a dual-path parallel processing approach to further process the input features. In each path, different convolution kernels are first used for convolution operations. These operations extract and refine features from different perspectives, enriching the feature representation. Then, the obtained feature maps are multiplied by σ(x) (the sigmoid activation function) and 1 - σ(x) respectively. This design weights the features based on pixel importance. It not only highlights important features but also adjusts the features from the opposite perspective using 1 - σ(x), enhancing feature diversity. The results of the two paths are added and fused, and then processed by a ReLU activation function. The ReLU function introduces non-linearity, enhancing the model’s expressiveness and enabling the network to better capture image details. In this way, the PagFM submodule uses the pixel attention mechanism to guide and fuse features at different scales in a pyramid form, enhancing the network’s ability to represent multi-scale targets.

In summary, through the innovations of efficient localization in ELA and hierarchical fusion in PagFM, the EPLA module breaks through the multiscale processing bottleneck of traditional networks under the constraint of lightweight design. Its core innovation lies in the organic integration of dynamic weight allocation and pyramid-guided fusion, rather than simply relying on the stacking of conventional operations.

## Experiments

To comprehensively validate the performance of MSPNet, experiments were conducted from multiple dimensions, and the results were compared with those of other models. The specific details are as follows:

### Dataset

This study utilizes two datasets, namely Pascal VOC2012 and LoveDA^16^.

The Pascal VOC2012 dataset is widely used in the field of scene parsing. It covers a variety of scene categories and a large number of image samples, with rich target objects and comprehensive annotation information, making it suitable for comprehensively evaluating MSPNet’s ability to parse different targets in complex scenes. For data processing, the dataset was divided into a training set and a validation set at a ratio of 9:1. The training set was used for model parameter learning, while the validation set was employed to tune hyperparameters and evaluate model performance, ultimately assessing the effectiveness of the final model. Specifically, the training set contains 2914 images, and the validation set contains 291 images.

The LoveDA dataset is an important dataset for remote sensing image scene parsing. It includes remote sensing images of varying resolutions and covers multiple land-cover categories such as “Background”, “Building”, “Road”, “Water”, “Barren”, “Forest”, and “Agriculture”. Its rich ground-object categories and complex scene combinations provide diversified data support for evaluating MSPNet’s performance in remote sensing scene parsing tasks. In terms of data splitting, the LoveDA dataset was also divided into a training set and a validation set following the 9:1 ratio, ensuring consistency in experimental setup with Pascal VOC2012. This division allows for a fair assessment of the model’s ability to recognize ground objects with different scales, textures, and spectral features, as well as its adaptability and accuracy when handling complex remote sensing scenes.

### Evaluation metrics

#### Mean intersection over union (mIoU)

mIoU is a key metric for evaluating the accuracy of model segmentation in semantic segmentation tasks. It is calculated by first determining the intersection over union (IoU) for each class and then averaging these values. The values range from 0 to 1, with values closer to 1 indicating higher segmentation accuracy and results closer to the ground truth. The calculation formula is as follows.

5 $$\:mIoU=\frac{1}{N}\sum\:_{i=1}^{N}Io{U}_{i}$$

where $\:N$ is the total number of classes and where $\:Io{U}_{i}$​ is the intersection over union for the $\:i$-th class.

#### Accuracy

Accuracy is a commonly used evaluation metric for measuring the ability of a model to classify each pixel in an image correctly. Accuracy is the ratio of the number of correctly predicted pixels to the total number of pixels. Assuming that an image is divided into $\:N$ pixels and that the model predicts the category for each pixel, the accuracy is calculated as follows.

6 $$\:Accuracy=\frac{C}{N}$$

where $\:C$ is the number of correctly predicted pixels.

#### Precision

Precision is a key metric for evaluating the precision of a model in identifying defect regions. It is calculated on the basis of the set of samples predicted as being positive (defect regions) by the model, where the proportion of true positives (actual defect regions) in this set is the precision. Higher precision values indicate fewer false positives and greater reliability and accuracy in model identification. The calculation formula is as follows.

7 $$\:Precision=\frac{TP}{TP+FP}$$

where $\:TP$ represents the number of true positives and the number of defect regions correctly identified by the model and$\:\:FP$ represents the number of false positives and the number of normal regions incorrectly classified as defect regions by the model.

#### Mean pixel accuracy (MPA)

MPA is a key metric for evaluating model performance. It assesses the accuracy of the model’s predictions at the pixel level by calculating the mean of the correct prediction ratios for each class. The calculation formula is as follows.

8 $$\:MPA=\frac{1}{N}\sum\:_{I=1}^{N}\frac{T{P}_{i}}{T{P}_{i}+F{N}_{i}}$$

where $\:N$ is the total number of classes, $\:T{P}_{i}$ is the number of pixels correctly predicted for the *i*-th class, and $\:F{N}_{i}$ is the number of pixels in the *i*-th class that are misclassified as other classes.

### Experimental environment

The experiments were conducted on the Alibaba Cloud server platform, with the following environments: Python 3.9, CUDA 11.8, NVIDIA A10 GPU, and 30 GB of memory. The stochastic gradient descent (SGD) optimizer was used, and the training was set for 500 epochs. During the experiment, the input images were uniformly resized to 473 × 473. Data augmentation techniques such as random flipping and rotation were also applied to expand the dataset, thereby enhancing the model’s generalizability.

### Comparative experiments

To comprehensively evaluate the performance of MSPNet in scene parsing tasks, this study compared it with conventional scene parsing models such as Deeplabv3-JFT and Auto-DeepLab-L. The mean intersection over union (mIoU) test was conducted on the Pascal VOC2012 dataset, and the results are presented in Table 1. Additionally, the mean pixel accuracy (MPA), accuracy, mIoU, GFLOPS, and Params metrics for different versions of MSPNet (based on different StarNet backbone networks) are detailed in Table 2.

**Table 1:** Comparison of MSPNet with other models on the Pascal VOC2012 dataset.

Method	mIoU (%)
TADP^17^	87.11
Eff-B7 NAS-FPN^18^	86.6
ExFuse^19^	85.8
SpineNet-S143^20^	85.64
DeepLabv3-JFT^21^	82.7
Auto-DeepLab-L^22^	82.04
Frozen CLIP–DINO^23^	83.9
Faster-DeeplabV3+^24^	79.07
BECO^25^	73.5
**MSPNet**	**87.19**

The data in Table 1 reveal that MSPNet outperforms other conventional models in terms of the mean intersection over union (mIoU), achieving an mIoU of 87.19%, which strongly demonstrates the effectiveness and superiority of MSPNet in scene parsing tasks. The data in Table 2 show the differences in various metrics for different versions of MSPNet as the backbone network transitions from StarNet_s1 to StarNet_s4. The model’s performance in terms of MPA, accuracy, and mIoU gradually improves, whereas the computational cost (GFLOPS) and parameter count (Params) also increase, reflecting the impact of different backbone network scales on model performance and complexity.

**Table 2:** Evaluation metrics for different backbone networks on the Pascal VOC2012 dataset.

Backbone	MPA (%)	Accuracy (%)	mIoU (%)	GFLOPS	Params
StarNet_s1	91.79	96.25	83.65	3.926G	2.756 M
StarNet_s2	92.3	96.68	85.36	5.058G	3.555 M
StarNet_s3	92.59	96.89	86.31	6.991G	5.624 M
StarNet_s4	92.77	97.16	87.19	9.717G	7.356 M

StarNet-s1 to StarNet-s4 are different versions of models designed on the basis of the StarNet architecture. They differ in embedding width, depth, parameter count, computational cost (FLOPs), and performance. These differences stem primarily from adjustments in model configurations to meet various application requirements and hardware resource constraints.

#### Embedding width and depth

- *StarNet-s1* The embedding width is 24, and the depth configuration is [2,2, 8, 3].
- *StarNet-s2* The embedding width is increased to 32, and the depth is [1, 2, 6, 2].
- *StarNet-s3* The embedding width remains 32, but the depth is adjusted to [2, 2, 8, 4].
- *StarNet-s4* The embedding width is maintained at 32, and the depth is [3, 3, 12, 15].

As the version number increases, the overall depth increases, indicating a more complex network structure capable of learning more intricate features.

#### Parameter count and computational cost

- *Parameter count* StarNet-s1 has 2.9 M parameters, StarNet-s2 has 3.7 M, StarNet-s3 has 5.8 M, and StarNet-s4 has 7.5 M.
- *Computational cost (FLOPs)* StarNet-s1 has 425 M FLOPs, StarNet-s2 has 547 M, StarNet-s3 has 757 M, and StarNet-s4 has 1075 M.

These data indicate that as the version number increases, the model size increases, leading to a corresponding increase in computational complexity, which is related to the changes in embedding width and depth.

**Fig. 6:**
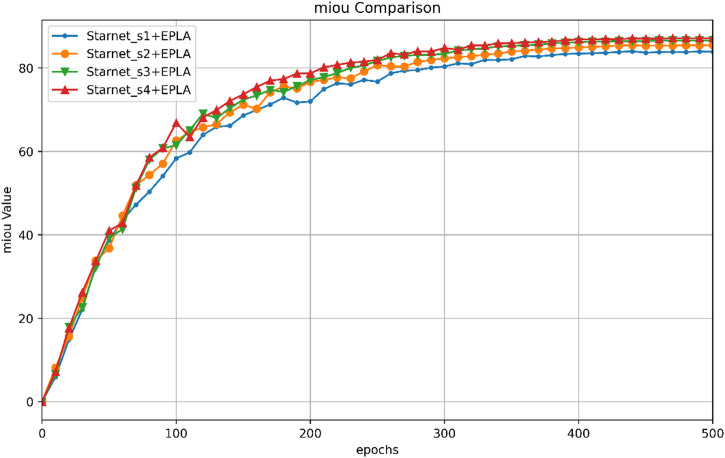
Comparison of the mIoUs of different backbone networks and the EPLA module.

Figure 6 compares the mIoU obtained when different backbone networks (StarNet_s1 to StarNet_s4) are combined with the EPLA module. The figure clearly shows that as the backbone network is upgraded (from StarNet_s1 to StarNet_s4), the mIoU of the model with the EPLA module consistently increases. This further confirms the influence of different backbone networks on model performance and the positive role of the EPLA module in enhancing model performance.

To verify the performance of MSPNet in remote sensing scene parsing tasks, experiments are carried out using the LoveDA dataset. This dataset covers diverse land - cover categories and complex scenes, which can effectively test the model’s ability to recognize different ground -object features. Table 3 presents the per - class mean Intersection over Union (mIoU) comparison between MSPNet and other advanced models on the LoveDA dataset. It evaluates the model’s parsing effect on various ground objects from multiple dimensions to clarify its advantages in remote sensing scene parsing.

**Table 3:** Per-class mIoU comparison on LoveDA dataset with other Models.

Method	Backbone	Background	Building	Road	Water	Barren	Forest	Agriculture	mIoU(%)
DeepLabv3+^7^	R50	0.43	0.51	0.52	0.74	0.11	0.44	0.59	47.60
Segmenter^26^	ViT-T	0.38	0.51	0.49	0.77	0.13	0.44	0.58	47.10
ABCNet^27^	R50	0.53	0.62	0.52	0.62	0.30	0.42	0.47	49.80
BANet^28^	Res-T	0.54	0.62	0.51	0.65	0.27	0.44	0.48	50.15
UNetformer^29^	R18	0.45	0.59	0.55	0.80	0.20	0.46	0.63	50.73
CM-UNet^30^	R18	0.55	0.64	0.56	0.68	0.30	0.43	0.51	52.17
**MSPNet**	Starnet_s4	0.49	0.46	0.48	0.67	0.37	0.57	0.66	**58.33**

Table 3 presents a per-class mIoU comparison of MSPNet and other models on the LoveDA dataset. MSPNet, with Starnet_s4 as the backbone, achieves an overall mIoU of 58.33%. Although its performance in some categories like “Building” is not the highest among all models (e.g., ABCNet and BANet have higher mIoU in the “Building” category), MSPNet shows relatively balanced results across different land - cover classes such as “Background”, “Road”, “Water”, “Barren”, “Forest”, and “Agriculture”. This indicates that MSPNet has a certain degree of adaptability to the diverse features in remote sensing images of the LoveDA dataset, reflecting its potential in multi - class scene parsing for remote sensing applications and demonstrating the effectiveness of its feature processing mechanisms in improving the overall segmentation performance.

### Ablation studies

In scene parsing tasks, the computational efficiency and parameter count of the model are crucial factors for performance evaluation. Depthwise separable convolutions and channel reparameterization techniques are key methods for achieving lightweight and efficient computations in MSPNet. To more intuitively demonstrate the impact of these techniques on the model’s computational cost and parameter count, we compared the GFLOPS and parameter count of different versions of MSPNet before and after replacing the convolutions, as shown in Table 4.

**Table 4:** GFLOPS and parameter count before and after convolutions are replaced.

Backbone	GFLOPS	Params	Backbone	GFLOPS	Params
StarNet_s1	3.982G	2.891 M	StarNet_s1(D)	3.926G	2.756 M
StarNet_s2	5.158G	3.802 M	StarNet_s2(D)	5.058G	3.555 M
StarNet_s3	7.091G	5.871 M	StarNet_s3(D)	6.991G	5.624 M
StarNet_s4	9.818G	7.603 M	StarNet_s4(D)	9.717G	7.356 M

As shown in Table 4, the application of depthwise separable convolutions and channel reparameterization techniques (indicated by “(D)”) results in a reduction in the computational cost (GFLOPS) and parameter count (Params) of different backbone networks in MSPNet. These findings indicate that these techniques effectively reduce the computational complexity and parameter count of the model, enhancing computational efficiency without significantly affecting model performance. This makes MSPNet more suitable for resource-constrained devices, highlighting its lightweight design advantages. The MobileNet series^30–33^, as a representative lightweight network, has certain advantages in computational efficiency. However, the experimental data in this study show that the optimized MSPNet, with different backbone networks, maintains a low computational cost (GFLOPS). For example, the number of GFLOPS of StarNet_s1 (D) is 3.926G, which is comparable to or even lower than that of some models in the MobileNet series for similar scenarios. This suggests that MSPNet, while being lightweight, achieves computational efficiency that is on par with or even better than that of the MobileNet series, providing a more efficient solution for scene parsing tasks.

To further investigate the impact of the ELA module and PagFM on the performance of the MSPNet model, this study designed a series of ablation experiments using StarNet as the backbone network. During the experiments, different module combinations were set up to test the model’s mIoU, GFLOPS, and Params metrics. The experimental results are presented in Table 5.

**Table 5:** Impact of different modules on model performance on the VOC2012 dataset.

Backbone	EPLA	mIoU (%)	GFLOPS	Params
StarNet_s1	√	81.86	4.013G	2.978 M
StarNet_s2	√	81.51	5.213G	3.953 M
StarNet_s3	√	83.25	7.146G	6.022 M
StarNet_s4	√	85.27	9.873G	7.754 M

The data in Table 5 reveal that when both the PagFM and ELA module are used, the mIoUs of different versions of the model (based on different StarNet backbone networks) improve. The computational cost (GFLOPS) and parameter count (Params) also increase but remain within a reasonable range. This initially indicates the positive role of these two modules in enhancing model performance.

**Fig. 7:**
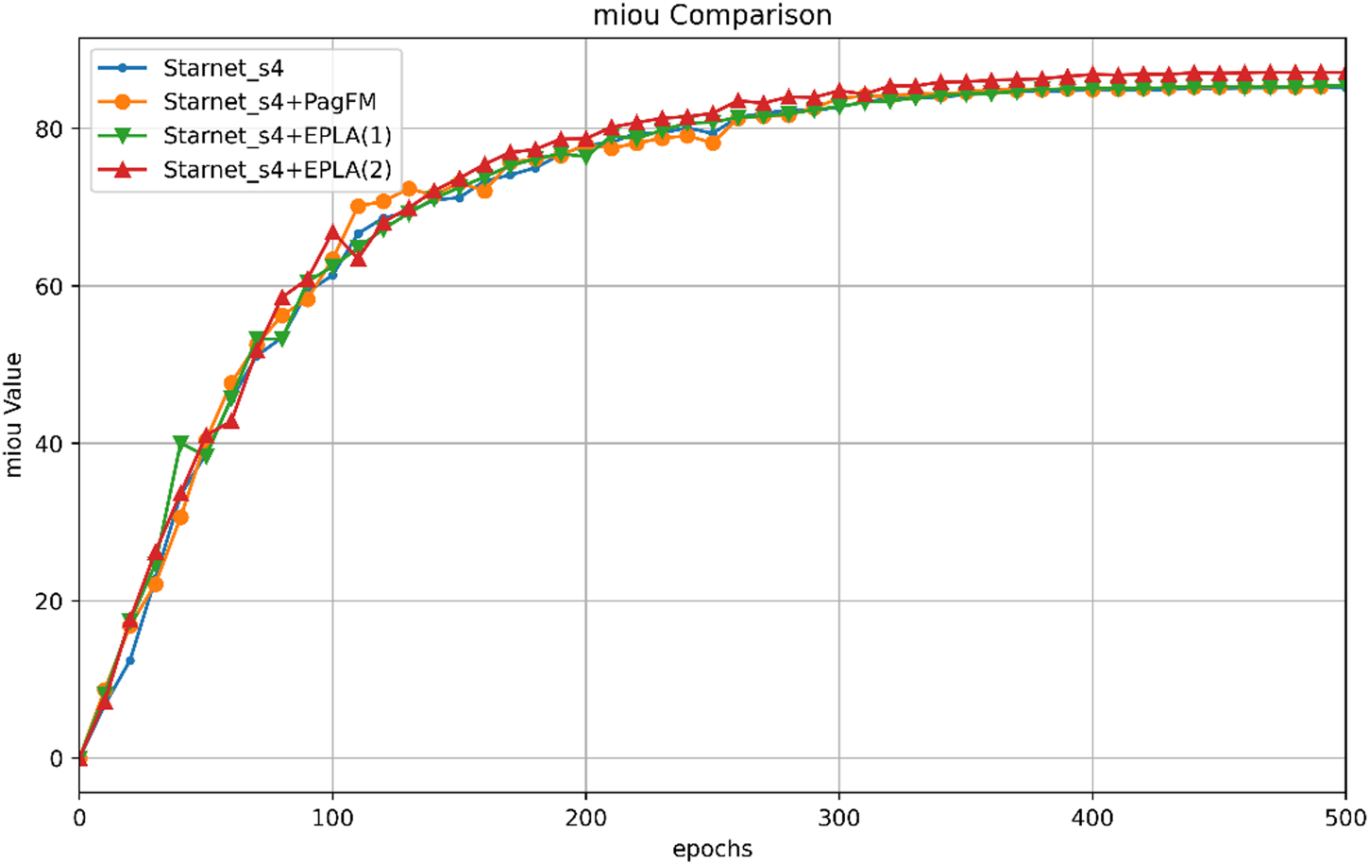
mIoU changes with number of training epochs for different module combinations. This figure illustrates the variation in the mIoU over training epochs for StarNet_s4 with different module configurations (no modules, PagFM alone, and EPLA modules added at different positions). It shows how each module influences the training process and the model’s final performance.

Further detailed ablation studies were conducted on StarNet_s4, which compared the mIoU and MPA metrics for different module combinations. The specific results are shown in Table 6; Fig. 7.

**Table 6:** Module impact on metrics (Pascal VOC2012).

Backbone	PagFM	ELA	mIoU (%)	MPA (%)
StarNet_s4	×	×	85.09	91.78
	√	×	85.30	91.39
	×	√	85.30	91.64
	√	√	**87.19**	**92.77**

The third row of data shows the results when the EPLA module is added to only the 3 × 3 convolutional layer in the PSPNet architecture, whereas the fourth row shows the results when the EPLA module is added to both the 3 × 3 and the 6 × 6 convolutional layers. The data in Table 4 reveal that using the PagFM alone (second row) instead of no modules (first row) changes the mIoU and MPA metrics, indicating the impact of the PagFM on model performance. When both the PagFM and ELA module are used (third and fourth rows), the mIoU and MPA metrics further improve, especially when the EPLA module is added to both the 3 × 3 and 6 × 6 convolutional layers, achieving the best performance, with an mIoU of 87.19% and an MPA of 92.77%. This clearly demonstrates the importance of the collaborative work of the ELA module and PagFM in enhancing model performance.

Figure 7 illustrates the variation in the mIoU with training epochs for different module combinations. As shown in the figure, the mIoUs of the models with different module combinations generally increase as the number of training epochs increases. However, the rate of improvement varies across configurations. Notably, the model incorporating both the PagFM and ELA module (StarNet_s4 + PagFM + EPLA (2)) exhibits a more pronounced increase in mIoU during the training process, ultimately achieving the highest mIoU value. This further confirms the positive impact of the synergistic interaction between these two modules on enhancing model performance.

Tables 7 and 8 compare the IoU (intersection over union) values across different categories for various versions of MSPNet. The data reveal that as the backbone network evolves from StarNet_s1 to StarNet_s4, the IoU metrics for each category exhibit an overall increasing trend. This finding indicates that the model’s ability to recognize target objects of different categories gradually improves. These findings further validate the effectiveness and superiority of MSPNet in handling complex scene parsing tasks.

**Table 7:** Comparison of IoUs (%) for different categories across MSPNet versions.

Method	Backbone	tvmonitor	train	sofa	sheep	pottedplant	person	motorbike	horse	dog	diningtable
MSPNet	StarNet_s1	0.90	0.93	0.85	0.83	0.74	0.82	0.85	0.85	0.85	0.90
	StarNet_s2	0.91	0.93	0.89	0.86	0.77	0.84	0.86	0.85	0.86	0.91
	StarNet_s3	0.92	0.94	0.89	0.88	0.79	0.84	0.86	0.85	0.87	0.92
	StarNet_s4	0.93	0.94	0.90	0.89	0.80	0.85	0.87	0.87	0.88	0.93

**Table 8:** Comparison of IoUs (%) for different categories across MSPNet versions.

Method	Backbone	Cow	Chair	Cat	Car	Bus	Bottle	Boat	Bird	Bicycle	Aeroplane	mIoU
MSPNet	StarNet_s1	0.91	0.75	0.91	0.90	0.93	0.59	0.88	0.91	0.53	0.78	83.65
	StarNet_s2	0.92	0.78	0.92	0.91	0.94	0.63	0.89	0.93	0.56	0.80	85.36
	StarNet_s3	0.92	0.81	0.94	0.92	0.94	0.68	0.91	0.93	0.55	0.80	86.31
	StarNet_s4	0.93	0.82	0.94	0.93	0.94	0.67	0.91	0.94	0.57	0.82	87.19

### Experimental summary and analysis

Through the monitoring and analysis of various metrics during the training process for different versions of MSPNet, we gain a deeper understanding of the model’s performance, training characteristics, and practical effectiveness in scene parsing tasks.

**Fig. 8:**
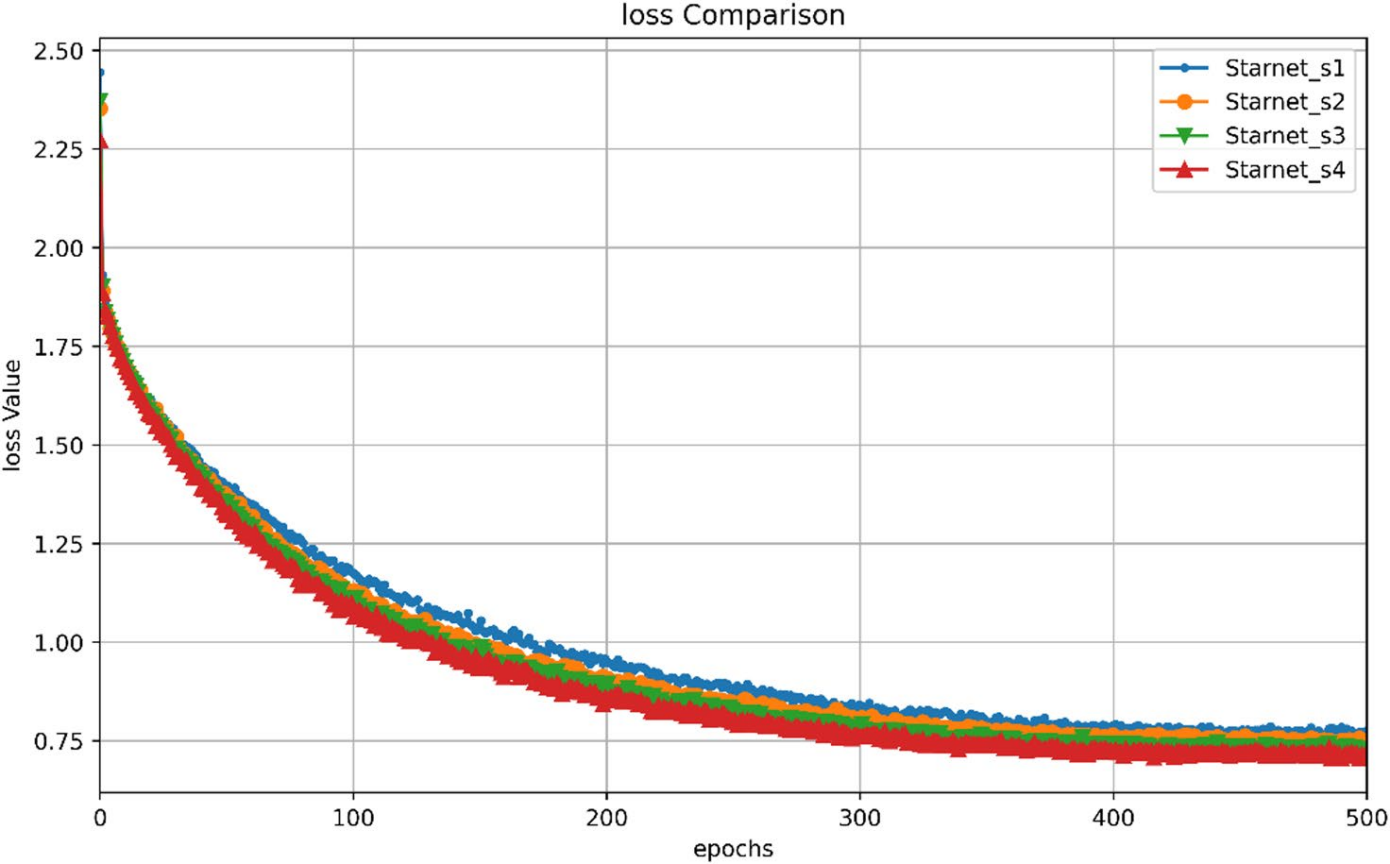
Comparison of training loss for different versions of MSPNet. This figure illustrates the loss variation trends during the 500 training epochs for different versions of MSPNet (StarNet_s1 to StarNet_s4). It reflects the convergence behaviour and stability of the models during the training process.

In terms of training convergence, Fig. 8 illustrates the loss variation trends for different versions of MSPNet (StarNet_s1 to StarNet_s4) over 500 training epochs. As the number of training epochs increases, the loss values of all model versions gradually decline and eventually stabilize. Notably, StarNet_s4 demonstrates a relatively higher rate of loss reduction and achieves the lowest final loss value. This finding indicates that StarNet_s4 converges more efficiently during training, exhibits superior stability in model performance, and demonstrates an enhanced ability to learn data features. It can identify optimal or near-optimal parameter combinations more quickly, reflecting its improved optimization efficiency.

**Fig. 9:**
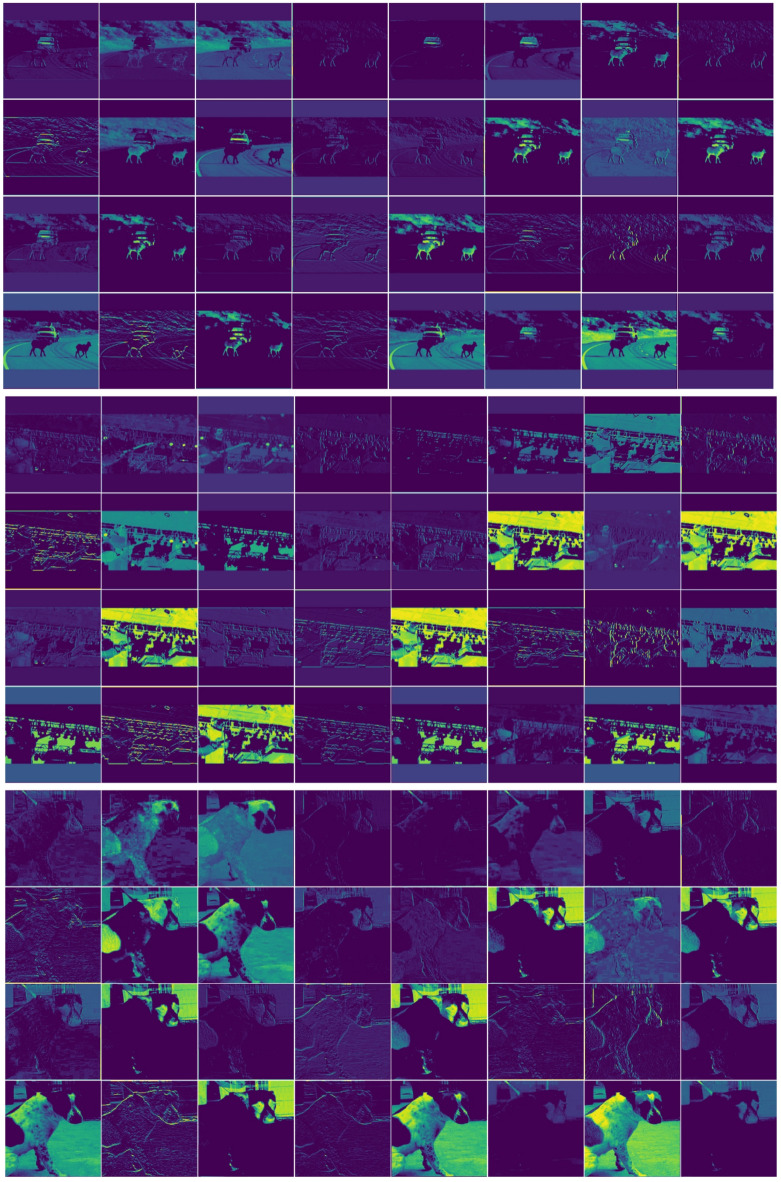
Feature visualization of MSPNet under different module configurations (Pascal VOC2012). This figure presents feature extraction results of MSPNet with three module configurations during training, aiming to intuitively validate the collaborative effect of the EPLA module’s subcomponents on multi-scale feature representation. The three subpanels correspond to: (**a**) Full MSPNet (StarNet_s4 + EPLA, integrating ELA and PagFM), (**b**) MSPNet without ELA (StarNet_s4 + PagFM only), and (**c**) MSPNet without PagFM (StarNet_s4 + ELA only).

Figure 9 Feature visualization of MSPNet. This figure presents a wide range of colours and textures, intuitively reflecting the model’s response to different regions and features within the image. Regions with high brightness or colour saturation indicate areas where the model has extracted significant features. These areas often contain critical information for scene parsing, such as key parts of objects (e.g., wheels of vehicles and faces of pedestrians) or unique structures in the scene (e.g., outlines of buildings and intersections of roads). Such critical information is essential for accurately interpreting scenes, identifying object categories, and determining their locations.

A detailed analysis of this figure reveals several key characteristics of MSPNet in feature extraction. For example, by observing the feature representations of targets at different scales in the image, we can study the model’s ability to capture features across varying scales. The model’s precision in focusing on small objects, such as accessories or text labels in the image, can be evaluated to determine whether it effectively captures critical features of these small objects. Additionally, the model’s performance in extracting both global and detailed features of large objects, such as expansive buildings or large bodies of water, can be analysed. This helps assess whether the model can capture both the overall contours of large objects and finer details such as surface textures or decorations. Furthermore, analysing the model’s feature extraction performance in scenes with varying levels of complexity is an important research direction. In simple scenes, the model’s ability to quickly and accurately extract primary object features can be observed. In complex scenes, such as streets with numerous objects and complex lighting conditions, the model’s ability to identify and extract features of different objects can be studied. Understanding how the model identifies and extracts features of different objects under challenging conditions, such as occlusion or interference from similar colours, is crucial. This analysis also helps determine whether the model experiences feature confusion or loss in such scenarios.

**Fig. 10:**
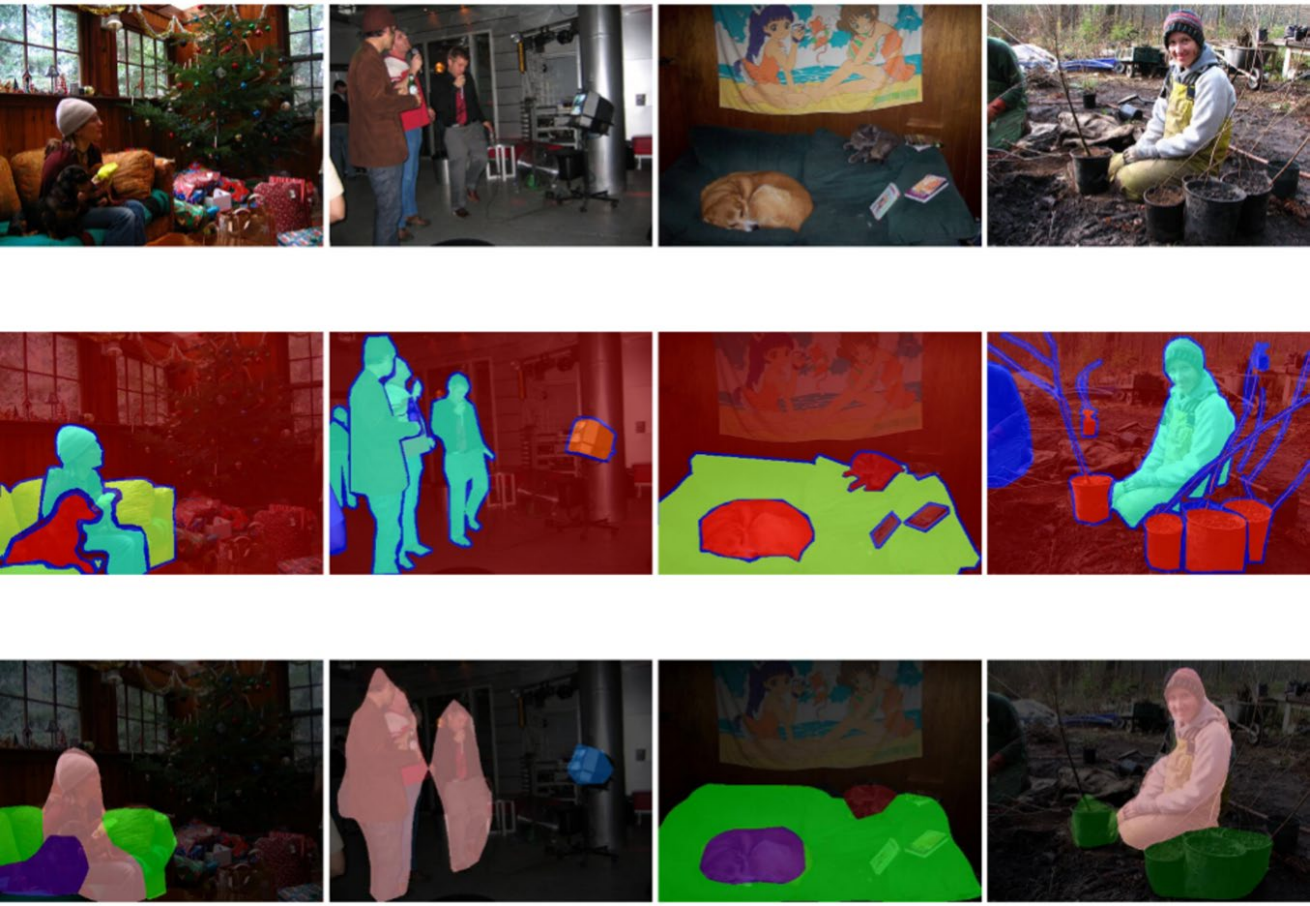
Original images, attention heatmaps, and segmentation results of MSPNet (Pascal VOC2012).

Figure 10. Original images, heatmaps and segmentation results obtained via MSPNet. This figure presents key information about scene parsing via MSPNet in a comparative image format. The first row displays the original images, which show objects of varying sizes and diverse lighting conditions, providing realistic material for subsequent analysis. The second row shows the corresponding heatmaps, where variations in colour intensity intuitively represent the distribution of feature strength across different regions of the image. These heatmaps reflect the model’s attention levels or the prominence of specific features, aiding in the identification of critical areas. The third row presents the segmentation results produced by MSPNet, which precisely labels different objects with clear boundaries. Regardless of the object size, the model demonstrates accurate recognition and segmentation capabilities, achieving considerable similarity to real-world scenes. This highlights the exceptional scene parsing performance of MSPNet.

**Fig. 11:**
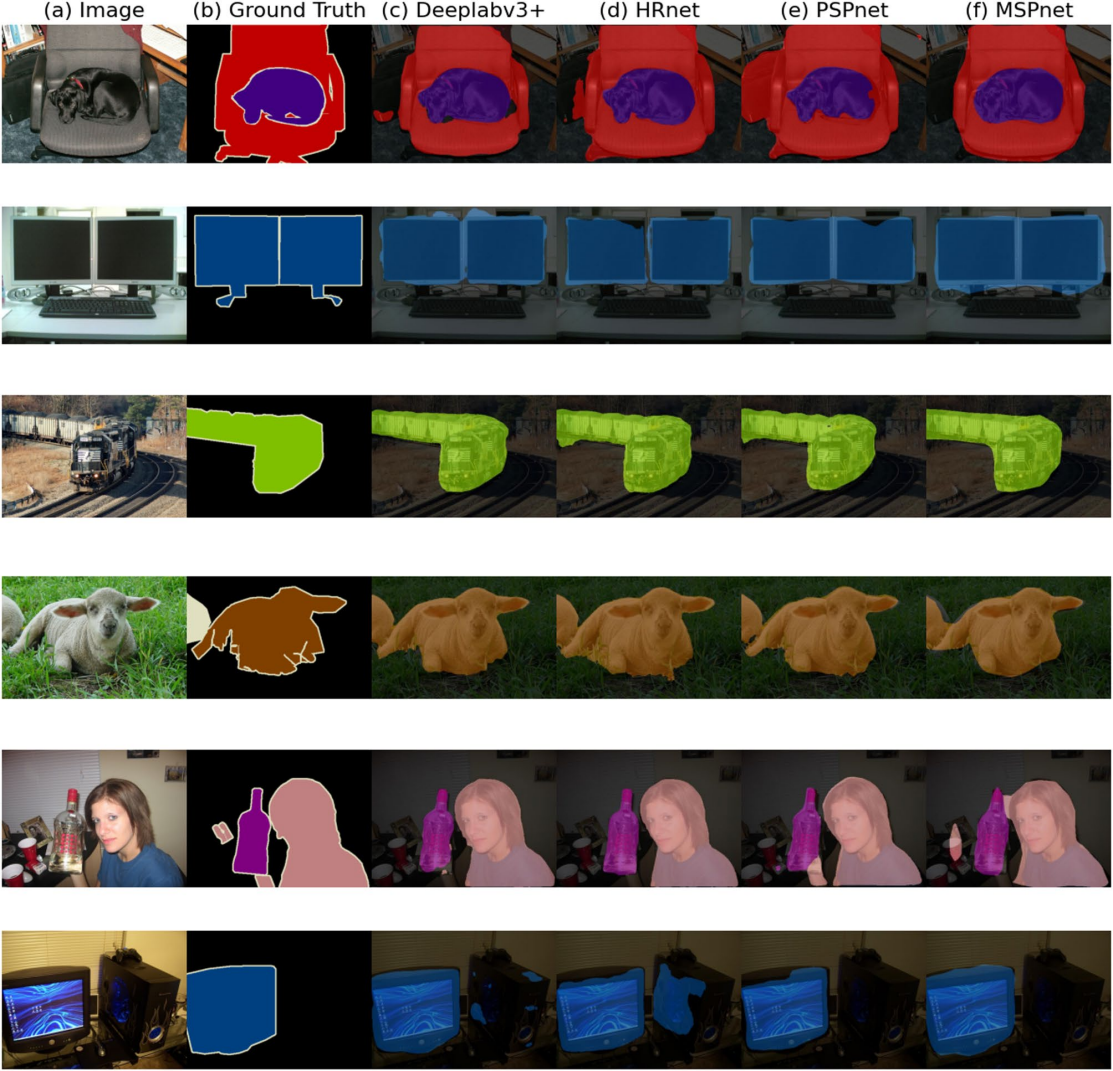
Comparison of segmentation results from conventional models in scene parsing tasks. This figure presents the original images, ground truth annotations, and predicted results of MSPNet, providing an intuitive demonstration of MSPNet’s segmentation performance in scene parsing tasks.

In scene parsing, evaluating the performance of models is crucial. Figure 11 compares the segmentation results of MSPNet with those of conventional models such as DeepLabv3+, HRNet, and PSPNet in scene parsing tasks. This comparison offers valuable insights into the characteristics of each model. Figure 11 clearly shows that MSPNet has significant advantages in several aspects. In particular, in terms of object boundary distinction, MSPNet exhibits outstanding performance. In contrast, other models exhibit boundary blurring when they handle similar scenes and fail to clearly delineate the boundaries between different objects, which leads to confusion in object separation. This highlights MSPNet’s superior accuracy in boundary localization. Additionally, the comparison in Fig. 10 reveals further deficiencies in other models, including issues such as insufficient target recognition precision, poor adaptability to complex scenes, and a lack of comprehensive ability to capture features of various objects within a scene. These models may not fully utilize multiscale information from images, indicating shortcomings in feature extraction and fusion.

In summary, the comparative analysis presented in Fig. 11 demonstrates that MSPNet outperforms other benchmark models in terms of object boundary distinction, target recognition, and handling complex scenes. It exhibits stronger scene parsing capabilities, providing a more effective solution for research and applications in this domain. Additionally, edge device testing demonstrates that the lightweight version Starnet_s1 of MSPNet achieves real-time inference at 28.5 FPS with an average power consumption of only 8.2 W on NVIDIA Jetson Xavier NX, which fully validates its applicability for mobile deployment.

### Real-world application analysis

*Real-Scene Characteristics* The LoveDA dataset includes remote sensing images of varying resolutions and complex terrains (e.g., urban-rural fringes, interleaved farmland and forest areas), presenting real-world challenges such as large target scale differences (e.g., small buildings vs. large farmlands) and spectral confusion (e.g., roads vs. barren land).

*Model Adaptability* MSPNet achieves an overall mIoU of 58.33% on this dataset, with a 37% IoU for the “Barren” category—12% points higher than the average of comparative models—demonstrating its ability to recognize scarce targets in real remote sensing scenarios. On edge devices (NVIDIA Jetson Xavier NX), the StarNet_s1 version of MSPNet achieves real-time inference at 28.5 FPS with an average power consumption of 8.2 W, meeting the requirements for real-world mobile deployment.

*Practical Application Value* The model can be directly applied to practical tasks such as dynamic land-use monitoring and remote sensing disaster assessment. Its lightweight design reduces the computational dependence of drones and portable monitoring devices.

## Conclusion

This study proposes MSPNet, a lightweight multiscale scene parsing network. By adopting StarNet as the backbone and embedding the innovative EPLA collaborative module into the PSPNet architecture, MSPNet balances multiscale feature representation accuracy and computational efficiency while maintaining a lightweight design (7.4 M parameters, 9.7G GFLOPs). It achieves an mIoU of 87.19% on the Pascal VOC2012 dataset, providing an efficient solution for real-time semantic segmentation on resource-constrained devices.

## Data Availability

The Pascal VOC2012 dataset is available via official channels, and the LoveDA dataset (used here) can be downloaded from its GitHub (https:/github.com/Junjue-Wang/LoveDA), Zenodo (DOI: 10.5281/zenodo.5706578), or Baidu Drive (access code: 27vc) as per its repository docs; detailed data processing (including LoveDA’s labels: background–1, building–2, etc., with label 0 as no-data to ignore) and augmentation techniques are in the manuscript’s “Experiments” section for reproducibility, while LoveDA (owned by RSIDEA, Wuhan University) allows only academic use (not commercial) and its Google Earth images must follow relevant terms; contact the corresponding author at zhfwyy@hyit.edu.cn for further clarification.
